# Thigh muscle co-contraction patterns in individuals with anterior cruciate ligament reconstruction, athletes and controls during a novel double-hop test

**DOI:** 10.1038/s41598-022-12436-6

**Published:** 2022-05-19

**Authors:** Ashokan Arumugam, Charlotte K. Häger

**Affiliations:** 1 Department of Physiotherapy, College of Health Sciences, University of Sharjah, P.O.Box: 27272, Sharjah, United Arab Emirates; 2 Department of Community Medicine and Rehabilitation – Physiotherapy Section, Umeå, University, 901 87 Umeå, Sweden

**Keywords:** Health care, Therapeutics, Rehabilitation

## Abstract

Efficient neuromuscular coordination of the thigh muscles is crucial in maintaining dynamic knee stability and thus reducing anterior cruciate ligament (ACL) injury/re-injury risk. This cross-sectional study measured electromyographic (EMG) thigh muscle co-contraction patterns during a novel one-leg double-hop test among individuals with ACL reconstruction (ACLR; *n* = 34), elite athletes (*n* = 22) and controls (*n* = 24). Participants performed a forward hop followed by a 45° unanticipated diagonal hop either in a medial (UMDH) or lateral direction (ULDH). Medial and lateral quadriceps and hamstrings EMG were recorded for one leg (injured/non-dominant). Quadriceps-to-Hamstring (Q:H) ratio, lateral and medial Q:H co-contraction indices (CCIs), and medial-to-lateral Q:H co-contraction ratio (CCR; a ratio of CCIs) were calculated for three phases (100 ms prior to landing, initial contact [IC] and deceleration phases) of landing. We found greater activity of the quadriceps than the hamstrings during the IC and deceleration phases of UMDH/ULDH across groups. However, higher co-contraction of medial rather than lateral thigh muscles during the deceleration phase of landing was found; if such co-contraction patterns cause knee adduction, a putative mechanism to decrease ACL injury risk, during the deceleration phase of landing across groups warrants further investigation.

## Introduction

Anterior cruciate ligament (ACL) tear is a common orthopedic injury^1^. The age at which individuals commonly suffer an ACL injury ranges from 5 to 64 years^2,3^ and most ACL injuries occur in sporting activities^4^. Following injury, risk of a secondary ACL tear increases by > 20%^5^. ACL injuries often result in long rehabilitation periods and might be career-ending or cause a long time lay-off from sports participation for athletes^2,6–8^. For sportspeople, only 30% of ACL injuries occur due to direct contact, while the remaining 70% occur in non-contact mechanisms^9–11^. Non-contact ACL injuries most often occur shortly after the initial foot contact (~ first 50 ms) during one and/or two-leg landing with relatively low knee (< 40°) flexion and multiplanar loading with frequent knee abduction^12–17^.

Incorporation of functional physical performance tests such as one-leg landing and/or cutting, mimicking sporting activities, is necessary in the assessment and training of ACL-injured persons. Functional physical performance tests are quantitative measures that emulate certain components of sporting activities. They have been shown to predict the risk of acquiring lower limb (sport) injuries and determine a player’s preparedness to return-to-play^18,19^. Such tests determine an individual’s ability to integrate joint stability, neuromotor control, flexibility, muscle strength, power, proprioception, agility, or balance into a functional task, mimicking a sports situation^18,19^. One-leg hop tests can be quantified using biomechanical tools such as motion capture systems to identify potential asymmetries between injured and uninjured legs in persons with a unilateral ACL injury, and can thereby be used to determine progress in rehabilitation, as well as assess ACL (re)injury risk^18,20,21^. There is however a lack of consensus as to the most optimal design of one-leg hop test best suited for such purposes. One-leg lateral^22^ and medial hops^23^ in particular emulate the movement patterns and forces occurring during side-step and crossover cutting maneuvers, respectively, to some extent. Moreover, medial hop tests with added visual-cognitive tasks have been reported to be reliable and, at the same time, challenge hop performance in healthy individuals^24^. When cutting maneuvers are performed in an unanticipated manner compared to pre-planned maneuvers, the time available for making optimal postural adjustments before task performance is decreased. Such maneuvers emulate sports-specific scenarios and have ecological validity^25^. This may further add to the ACL strain due to an increase in internal/external rotation and varus/valgus movement in the knee^26,27^. Aberrant motion patterns of the lower limb relating to ACL injury risk are often investigated during the deceleration phase of one-leg landing (initial 50% of stance)^28,29^. Knee abduction and internal rotation angles and (external) moments have been reported to be increased during the unanticipated one-leg cutting maneuvers compared to the anticipated counterparts^30^. Therefore, we have previously designed a novel one-leg double-hop test consisting of a forward hop followed by an unanticipated diagonal hop in a medial or lateral direction involving cutting maneuvers. Our test requires movement control and full loading with the leg being tested and prevents compensation from the contralateral leg during the land-and-cut maneuver. This might help to quantify/discriminate dynamic limb motion asymmetries in individuals with ACL injury/reconstruction and healthy-knee controls. Moreover, the test is easy to administer (if transferred to a more user-friendly clinical version), less time-consuming, requires very little space compared to a run-and-cut task and allows knee-challenging unanticipated change of direction with one-leg landing. This test has been found to reliably capture hip and knee angles and moments in healthy individuals^31^ and in those with ACL reconstruction (ACLR)^32^. In the current paper, we further investigate our unanticipated double hop test by determining thigh muscle co-contraction patterns during the pre-landing, initial (foot) contact (IC), and deceleration phases, as the latter two are the most vulnerable phases for non-contact ACL injuries. We hypothesized that thigh muscle activation patterns augmenting or mitigating ACL injury risk are evident during the one-leg double-hop test with an unanticipated change of direction.

Neuromuscular control of the thigh muscles alters knee stability by controlling motion and loading of the knee joint^33^ during dynamic tasks. An increased electromyographic (EMG) activity of the quadriceps compared to that of the hamstrings has been reported during one-legged landing in healthy athletes^34^ which results in an anterior translation of the tibia relative to the femur and increases ACL strain^35,36^. Thus, a balanced co-contraction of the quadriceps and hamstrings prior to one-leg landing is crucial in order to prepare for landing^37^. Conversely, quadriceps activation failure (bilateral)^38^ and weakness^39^ have been demonstrated in individuals with a unilateral ACLR or deficiency (managed conservatively) compared to healthy controls. Increased coactivation of the hamstrings during maximal or submaximal isometric knee extension in individuals with ACLR or ACL deficiency compared to healthy controls might restrict anterior tibial translation and improve joint stability^39,40^. This further reflects a protective strategy, based on an ACL-hamstring synergy ^40^, adopted by these individuals to mitigate ACL injury risk. A recent systematic review found no differences in quadriceps and hamstrings activity onset prior to one-leg landing or decelerating tasks between individuals with ACL injury or ACLR and healthy controls^41^. However, the included studies (*n* = 5) in that review were of poor to moderate quality^41^ and the outcomes of such studies must be interpreted cautiously.

A differential recruitment of medial versus lateral thigh muscles has been reported during different tasks in healthy individuals^34,42,43^ and persons with knee osteoarthritis^44^. A discordant co-contraction between the medial and lateral thigh muscles, where the medial muscles show lower co-contraction than the lateral group, limits the ability to resist abduction load and eventually abducts the knee and increases ACL strain^45,46^. Neuromuscular training has been reported to increase medial hamstring activation and might help in preventing the knee from excessive abduction^47^, a mechanism to lower the risk of ACL injury.

Activity level, rather than ACL injury, might be a contributing factor for proprioceptive deficits^48^. Moreover, previous studies have indicated differences in neuromuscular contributions (EMG-moment relationships) to dynamic knee stability^49^ and altered thigh muscle co-activation patterns during knee joint positions tests^50^ between individuals with and without ACL injury. We expected that our participants with ACLR would have different physical activity levels (light labor to competitive sports based on Tegner scores). Whether differences in physical activity levels and ACL injury status affect thigh muscle co-contraction patterns during our novel test with an unanticipated change of direction need further investigation. Therefore, the aim of this study was to examine EMG co-contraction patterns of the hamstrings and quadriceps muscles during our unanticipated one-leg double-hop test. Specifically, the study aimed to determine whether co-contractions of these muscle groups differ during the pre-landing and deceleration phases of the land-and-cut maneuver in three groups (individuals with ACLR, [healthy-knee] elite athletes and controls) of mixed or different physical activity levels (sedentary to competitive sports). Our hypotheses were three-fold: (1) all groups would present with high Quadriceps-to-Hamstring (Q:H) ratios in the pre-landing phase and low Q:H ratios in the IC and deceleration phases of unanticipated medial diagonal hop (UMDH) or unanticipated lateral diagonal hop (ULDH) which would emulate an ACL injury risk situation; (2) there would be a significant interaction between groups and phases of landing for medial and lateral Q:H co-contraction indices (CCI) for UMDH/ULDH; (3) all groups would present with a low medial-to-lateral Q:H co-contraction ratio (CCR, a ratio of CCIs) for the pre-landing phase and a high medial-to-lateral Q:H CCR for the IC and deceleration phases for UMDH/ULDH.

## Methods

### Study design and setting

This cross-sectional study was conducted at the U-motion laboratory, Department of Community Medicine and Rehabilitation, Umeå University, Sweden. The study was ethically approved by the Regional Ethical Review Board in Umeå (reference: 2015/67–31). All participants provided their prior written informed consent.

### Participants

Three groups of participants (*n* = 80) aged between 17 and 34 years were recruited for this study: (1.) 34 individuals (25 females) who had undergone unilateral ACLR 33.7 ± 32.2 months prior, (2.) 22 asymptomatic elite athletes (19 females) who were regularly involved in neuromuscular training of the knee and actively playing in the highest or second highest league in their respective sport, and (3.) 24 asymptomatic non-athletic controls (20 females) who were not performing any specific knee-demanding activities, resistance exercises or participating in group training activities were considered acceptable. All groups were recruited through convenience sampling via adverts posted around the university campus, emails, and word of mouth. Individuals with ACLR were recruited, regardless of their physical activity level, if they were at the end phase of rehabilitation or had completed rehabilitation and were able to perform hops on the ACLR leg without any pain or discomfort. Participants with any history of hip, knee or ankle injuries within the past 6 months (other than ACL injury for ACLR participants) or diagnosed with any ongoing musculoskeletal, rheumatic, or neurological diseases were excluded. Each participant was examined by an experienced physiotherapist prior to the test sessions to ensure their eligibility.

Self-reported knee function and physical activity levels of all participants were measured using the following questionnaires: Lysholm questionnaire, Knee injury and Osteoarthritis Outcome Score (KOOS), Tegner score, International Physical Activity Questionnaire (IPAQ)—short form, and the International Knee Documentation Committee (IKDC) form. In addition, quadriceps and hamstring isometric strength was assessed using the Kin-Com® dynamometer (Kinetic communicator 125 Auto Positioning, Chattanooga Group Inc.; Hixon, TN, USA).

### Instrumentation

A Noraxon TeleMyo™ Direct Transmission System (DTS) Belt Receiver (Noraxon Inc., USA), four DTS EMG transmitters, adhesive disposable silver/silver chloride surface electrodes, a 3-dimensional motion analysis system (Oqus, Qualisys, Sweden) with eight high-speed cameras, and two floor-embedded force plates (Kistler Winterthur, Switzerland; sampling-frequency: 1680 Hz) were used. The details of marker set configuration (56 retroreflective markers) and motion analysis are elaborated in our previous reliability studies on the one-leg double-hop test^31,32^.

### Procedure

Relevant demographic and anthropometric data were collected before testing. Each participant’s self-preferred leg to kick a ball was noted as their dominant leg. All the participants performed the tests barefoot and wore a sports bra and/or tight training shorts.

### Electromyography

The recommendations from the Surface Electromyography for the Non-Invasive Assessment of Muscles (SENIAM) committee were followed for recording muscle activity^51^. Participants’ skin over the recording site was shaved, abraded with sandpaper and swabbed with antiseptic wipes (75% isopropyl alcohol). Then Ag/AgCL surface electrodes were placed at an inter-electrode distance of 2 cm on the biceps femoris, medial hamstring, vastus lateralis and vastus medialis (Table 1) of one leg (ACLR group: the injured leg; control/athlete group: non-dominant leg). We chose the nondominant legs of healthy-knee athletes and controls to provide a more stringent comparison with the injured legs of ACL groups^52–55^. EMG data were recorded at 1500 Hz with Noraxon Telemyo 2400t G2 telemetric system (Noraxon Inc., Scottsdale, AZ, USA) using QTM software (Qualisys, Inc.).

**Table 1:** Surface electromyography–electrode placement guidelines.

Muscle	Electrode placement*	Electrode alignment*
Biceps femoris	50% on the line between the ischial tuberosity and the lateral epicondyle of tibia	In the direction of the line between the ischial tuberosity and the lateral epicondyle of the tibia
Medial hamstring	50% on the line between the ischial tuberosity and the medial epicondyle of tibia	In the direction of the line between the ischial tuberosity and the medial epicondyle of the tibia
Vastus lateralis	2/3 on the line from the anterior superior iliac spine to the lateral side of the patella	In the direction of the muscle fibers
Vastus medialis	80% on the line between the anterior superior iliac spine and the joint space in front of the anterior border of the medial collateral ligament of the knee	Almost perpendicular to the line between the anterior superior iliac spine and the joint space in front of the anterior border of the medial collateral ligament of the knee

*Guidelines retrieved from SENIAM (www.seniam.org).

### One-leg double-hop test

**Figure 1:**
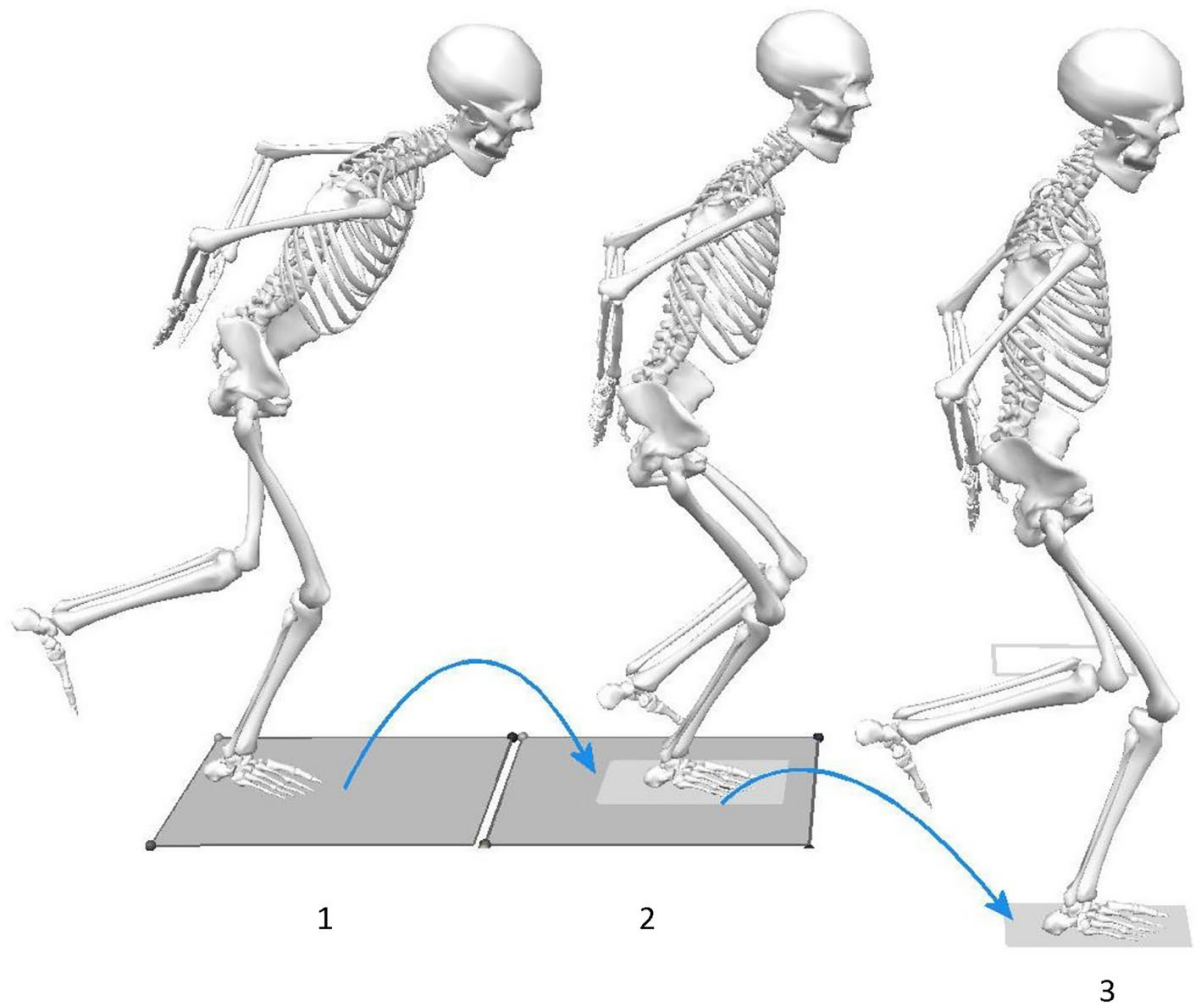
A novel unanticipated double hop performed with the right leg: 1. right foot planted on the 1st force plate; 2. forward hop landing of the right leg on the 2nd force plate at a distance of 25% of their height by reacting to a light signal indicating the target areas (rectangle boxes) to land and the subsequent direction of hopping (in a random order); 3. diagonal hop landing of the right leg in the lateral direction at an estimated angle of 45°and at a distance of 25% of their height. Images were captured from Visual 3D software (v5.02.30, http://www.c-motion.com) and then edited with Corel PaintShop Pro X9 (v19.2.0.7, http://www.paintshoppro.com).

The procedure was verbally explained to the participants at the start of the test session. Our one-leg double-hop test comprised of a forward hop followed by an immediate diagonal hop 45° to the medial or lateral direction in an unanticipated manner (see Fig. 1 and below for a more detailed description)^31,32^. Following two practice trials for each leg, participants were required to perform a minimum of 12 successful unanticipated diagonal hops (three per direction [medial/lateral] per leg). Inclusion of three trials for functional tests has been recommended by the IKDC^56^.

Participants stood on one foot on a force plate with their hands holding a 20 cm long rope (with knots at both ends) behind their lower back. They hopped forward onto the second force plate, and then performed a cutting maneuver (diagonal hop) in either the medial or the lateral direction at an angle of 45°^57^ (Fig. 1) over a predetermined distance (25% of their body height) guided by the light signal and further indicated by an adhesive tape affixed on the floor.

As for unpredictability of the hop direction, a projector mounted in the ceiling provided the visual cue (illumination of rectangles on the floor at a distance of 25% of their body height) upon initiation of the hop, to indicate whether the participant should attempt to land in a position medial or lateral to the direction of the forward hop. The order of direction was pseudorandomized. The visual cue was triggered as soon as vertical ground reaction force (VGRF) on the first force plate fell below 80% of its peak value during the push-off phase of the forward hop. Participants were required to perform the cutting maneuvers as quick as possible after receiving the visual cue indicating the hop direction. While hopping with the right leg, cutting done to the right side was named as ULDH and the left side as UMDH.

The trials were declared successful by an assessor during testing and then verified afterwards by another assessor using video analysis and Qualisys data. A ULDH or UMDH was considered successful if the participants hopped in the appropriate direction, covered the required distance and maintained balance and control upon landing without touching down their other foot. The trials were declared unsuccessful if the participants hopped in the opposite direction (performed UMDH instead of ULDH or vice versa), paused^58^, touched the force plate with the contralateral foot upon landing, landed outside the target (illuminated) areas, had extra hops upon landing or hands let go of the rope. Participants were allowed repeat the test till a minimum of three successful trails was achieved.

Two events based on vertical ground reaction force (VGRF) on the second force plate were used to define the stance phase of landing and for extraction of EMG data during the timeframe of interest: IC, defined by an increase in VGRF by 20 N for the first time, to toe-off, marked by a fall in the VGRF below 20 N. EMG and force data were synchronized through a square wave transmitted by the Qualisys system.

### Data processing

EMG data were band-pass filtered between 20 and 500 Hz through a fourth order Butterworth filter and then Root Mean Square (RMS) filtered with a 20 ms sliding window^59^ to generate a linear envelope. Thigh muscles EMG activity of the hopping leg on the second force plate (Fig. 1) was averaged for the following phases: 100 ms prior to initial foot contact^60^ (pre-landing phase), first 50 ms of foot contact^61^ (IC), and IC to peak knee flexion^62^ (deceleration phase) of the land-and-cut maneuver.

The peak EMG value observed during landing^46^, i.e., between the pre-landing and the end of the land-and-cut maneuver was used to normalize linear envelopes at each phase of interest. An average of three successful trials for each participant (with the shortest stance time on the second force plate) was included in the analysis.

Mean EMG activity of the vastus lateralis and vastus medialis was divided by that of the medial hamstrings and biceps femoris muscles to calculate Q:H ratio. In addition, muscle CCI was defined as the concurrent activation of two muscle groups: EMG_S_/EMG_L_*(EMG_S_ + EMG_L_)^63^ where EMG_S_ is the EMG magnitude of the less active muscle and EMG_L_ is the EMG magnitude of the more active muscle. This equation was applied for each data sample and the resulting curve was integrated for the (three) phases of interest^63^. A high CCI can be interpreted as a high level of muscle activity in both muscles while low CCI would imply either low activity in both muscles or that one of the muscles has high activity and the other has a low activity^63^. CCIs were calculated for the three landing phases of interest for the following muscle pairs: vastus lateralis and biceps femoris (lateral Q:H CCI), and vastus medialis and medial hamstrings (medial Q:H CCI). To determine whether CCIs were balanced between the medial and lateral sides, the medial-to-lateral Q:H co-contraction ratio (CCR) was calculated by dividing the medial Q∶H CCI by the lateral Q∶H CCI^42^.

### Statistical analysis

Data were tested for normal distribution using the Shapiro–Wilks tests. Skewed data were log-transformed and subjected to parametric analysis given that the log-transformed data followed normal distribution. Multiple 3 (groups) × 3 (phases) mixed analysis of variance models (ANOVAs) were used with group as a between-subject factor and phase of landing as a within-subjects factor for each hop direction (UMDH/ULDH). Kruskal–Wallis tests were used to test between-group mean differences in participant characteristics as those data were skewed, except for normally distributed thigh muscle strength values subjected to one-way ANOVA. Post hoc multiple tests were adjusted with a Bonferroni correction. The level of significance was set at *p* < 0.05. For all statistical analyses, the Statistical Package for the Social Sciences (Version-27, IBM SPSS Statistics, USA) was used.

## Results

Demographic and anthropometric characteristics of all participants and ACL injury-related data are summarized in Table 2. There was a significant difference in age between the ACLR and athlete groups (*p* = 0.020) where athletes were slightly younger than those with ACLR. Also, individuals with ACLR had greater knee laxity (*p* < 0.001) than controls and athletes when measured with KT1000 arthrometer (Table 2). Isometric peak torque of the quadriceps and hamstrings were not significantly different between the groups.

**Table 2:** Participant characteristics.

Mean (SD)	ACLR	Controls	Athlete	Kruskal–Wallis test (*p* value)	ACLR vs. Controls*	ACLR vs. Athletes*	Controls vs. athletes*
*Participants (n)*	34	24	22	–			
Males/females	9/25	4/20	3/19	0.446**			
Age (years), *mean (SD)*	24.5 (4.6)	23.4 (3.4)	21.3 (2.9)	**0.020**	1.000	**0.022**	0.097
**Anthropometric measurements mean (SD)**							
Height (cm)	171.9 (8.1)	169.8 (6.9)	171.6 (7.4)	0.884	–	–	–
Body mass (kg)	70.6 (11.1)	64.4 (7.0)	66.2 (9.0)	0.130	–	–	–
Body mass index (kg/m^2^)	23.8 (2.5)	22.3 (2.1)	22.4 (1.9)	**0.029**	0.090	0.065	1.000
A/ND KT1000-15^۴^	2.42 (1.52)	0.29 (0.71)	0.21 (0.79)	** < 0.001**	** < 0.001**	** < 0.001**	1.000
A/ND KT1000-20^۴^	2.74 (1.60)	0.45 (0.75)	0.19 (0.84)	** < 0.001**	** < 0.001**	** < 0.001**	1.000
A/ND KT1000-30^۴^	2.79 (1.86)	0.27 (0.93)	0.24 (0.91)	** < 0.00**1	** < 0.001**	** < 0.001**	1.000
Quadriceps peak torque (Nm/Kg)^	2.56 (0.55)	2.35 (0.58)	2.51 (0.43)	0.324***	–	–	–
Hamstring peak torque (Nm/Kg)^	1.05 (0.23)	1.11 (0.23)	1.11 (0.23)	0.500***	–	–	–
Duration post-injury (months)^#^	40.6 (35.5)	–	–	–	–	–	–
Duration post-surgery until test (months)^#^	18 (7–129)^@^	–	–	–	–	–	–
*Injured leg*							
Dominant	23	–	–	–	–	–	–
Non-dominant	11	–	–	–	–	–	–
Sports-related Injuries^$^	33	–	–	–	–	–	–
Road trauma	1						

Significant values are in [bold].

A, limb with anterior cruciate ligament reconstruction; ACLR, anterior cruciate ligament reconstruction; ND, non-dominant limb of the controls and athletes.

*Post-hoc comparisons (*p* vlaues) abjusted by Bonferroni correction for multiple tests.

**p value based on chi-square test.

***p values based on one-way ANOVA as these data were normally distributed; missing data for one athlete were omitted from analysis.

^۴^KT1000 arthrometer device was used to measure knee laxity at 15, 20 and 30 lb force; data represent differences between ACLR and noninjured knees (ACLR group) or dominant and non-dominant side knees (controls and athletes); missing data for one with ACLR, two controls and one athlete were omitted from analysis.

^Missing isometric strength data for one with ACLR and one athlete were omitted from analysis.

^#^Data missing for one participant with ACLR. ^@^Median (range).

^**$**^Mechanism of injury: 10 contact, 24 noncontact; Sport related to injury: 12 soccer, 10 floorball, 2 downhill skiing, 2 gymnastics, 2 rugby, 2 handball, 1 aerobic training, 1 mixed martial arts, 1 Kung-Fu.

Self-estimated knee function was lower for individuals with ACLR compared to controls and athletes (Table 3). Level of physical activity also differed between groups where athletes were more active than controls and those with ACLR (*p* ≤ 0.039) as estimated by IPAQ. Individuals with ACLR had a median Tegner score of 6 (range: 3–9) at the time of our study but their median pre-injury score was 9 (range: 3–10). All three groups differed significantly in activity level (athletes > ACLR > controls) according to the Tegner scores at the time of data collection. All but one person with ACLR were injured during sports participation and 23/34 (68%) had injured their dominant leg (Table 3) which was the right leg in 87% (20/23) of the cases.

**Table 3:** Self-reported knee function and physical activity level presented as median (range: min–max) for participants with ACLR, control and athlete groups.

Variables	ACLR (*n* = 34)	Controls (*n* = 24)	Athletes (*n* = 22)	Kruskal–Wallis test (*p* value)	ACLR vs. Controls**	ACLR vs. Athletes**	Controls vs. athletes**
Lysholm score*	86 (64–100)	100 (90–100)	100 (85–100)	** < 0.001**	** < 0.001**	** < 0.001**	1.000
KOOS subscales*							
Symptoms	82.1 (53.6–100)	100 (89.3–100)	100 (82.1–100)	** < 0.001**	** < 0.001**	**0.001**	1.000
Pain	91.7 (72.2–100)	100 (77.8–100)	100 (83.3–100)	** < 0.001**	** < 0.001**	** < 0.001**	1.000
Activities of daily living	100.0 (77.8–100)	100 (97.1–100)	100 (91.2–100)	**0.005**	**0.014**	**0.031**	1.000
Sports or recreational activities	85 (40–100)	100 (90–100)	100 (75–100)	** < 0.001**	** < 0.001**	** < 0.001**	1.000
Quality of life	68.8 (25–100)	100 (75–100)	93.8 (68.8–100)	** < 0.001**	** < 0.001**	** < 0.001**	0.686
Tegner score at present^†^	6 (3–9)	4 (1–6)	8 (8–9)	** < 0.001**	** < 0.001**	**0.006**	** < 0.001**
IPAQ^‡^	2866 (120–10,719)	2484 (693–7278)	4158 (2253–7758)	**0.001**	0.324	**0.039**	** < 0.001**
IKDC2000*	82 (64–100)	100 (90.8–100)	100 (66.7–100)	** < 0.001**	** < 0.001**	** < 0.001**	1.000
TSK^§^	30 (20–44)	–	–	–			

Significant values are in [bold].

ACLR, anterior cruciate ligament reconstruction; IKDC, International knee documentation committee; IPAQ, International Physical Activity Questionnaire; KOOS, Knee injury and Osteoarthritis Outcome Score; TSK: Tampa Scale for Kinesiophobia.

For all data in this table, data were missing for 2 participants (1 individual with ACLR and 1 athlete).

**Post-hoc comparisons (*p* vlaues) abjusted by Bonferroni correction for multiple tests.

^†^An activity scale ranging from 1 to 10 indicating current level of knee-demanding activities.

^**‡**^A scale of estimated metabolic equivalent (MET–minutes/week) from total duration of physical activities throughout the previous week with a high score indicating greater physical activity level.

*A scale ranging from 0 (worst) to 100 (best) indicating self-estimated knee function.

^§^A scale of fear of movement with scores ranging from 17 to 68 with a high score indicating more fear of movement.

### Q:H ratio

The IC phase rendered a higher Q-H ratio than the pre-landing and deceleration phases, regardless of group, for both directions (Table 4) owing to a significant main effect for phases (*p* < 0.001) for Q:H ratio (Table 5). There was no significant interaction between groups and landing phases for UMDH or ULDH.

**Table 4:** Median (interquartile range) values of thigh muscle co-contraction indices and ratios for UMDH and ULDH.

Variable	ACLR (*n* = 34)			Controls (*n* = 24)			Athletes (*n* = 22)		
Landing phases	Pre-100 ms	IC	Deceleration	Pre-100 ms	IC	Deceleration	Pre-100 ms	IC	Deceleration
**UMDH**									
Q:H ratio	0.54 (0.38)	3.09 (4.39)	1.81 (1.60)	0.52 (0.54)	3.56 (2.13)	2.33 (1.31)	0.51 (0.34)	3.90 (3.99)	2.78 (2.14)
Medial Q:H CCI	0.13 (0.11)	0.32 (0.21)	0.43 (0.16)	0.14 (0.09)	0.21 (0.21)	0.31 (0.16)	0.18 (0.08)	0.28 (0.30)	0.43 (0.18)
Lateral Q:H CCI	0.14 (0.11)	0.23 (0.26)	0.33 (0.27)	0.18 (0.15)	0.20 (0.11)	0.25 (0.10)	0.16 (0.12)	0.22 (0.25)	0.36 (0.33)
Medial-to-lateral Q:H CCR	0.89 (0.42)	1.36 (1.54)	1.29 (0.84)	0.74 (0.91)	1.34 (0.79)	1.25 (0.81)	1.28 (0.64)	1.60 (0.99)	1.22 (1.21)
ULDH									
Q:H ratio	0.48 (0.31)	2.12 (2.14)	1.38 (0.87)	0.38 (0.33)	2.28 (2.09)	1.85 (0.95)	0.37 (0.54)	1.83 (2.25)	1.72 (1.17)
Medial Q:H CCI	0.13 (0.11)	0.36 (0.20)	0.41 (0.16)	0.15 (0.09)	0.29 (0.27)	0.38 (0.17)	0.16 (0.09)	0.37 (0.33)	0.41 (0.24)
Lateral Q:H CCI	0.15 (0.09)	0.33 (0.19)	0.37 (0.19)	0.18 (0.12)	0.28 (0.22)	0.35 (0.19)	0.14 (0.14)	0.28 (0.23)	0.42 (0.26)
Medial-to-lateral Q:H CCR	0.96 (0.56)	1.01 (1.08)	1.05 (0.32)	0.93 (0.47)	1.18 (0.82)	1.08 (0.65)	1.24 (0.68)	1.36 (0.99)	1.23 (0.57)

ACLR, anterior cruciate ligament reconstruction; CCR, cocontraction ratio; Q:H, quadriceps to hamstring; Q:H CCI, quadriceps to hamstring cocontraction index; ULDH, unanticipated lateral diagonal hop; UMDH, unanticipated medial diagonal hop.

Landing phases: pre-100 ms = pre-landing phase, 100 ms prior to initial foot contact; IC = 50 ms after initial foot contact; deceleration phase = IC to peak knee flexion.

**Table 5:** Results of 3*3 (groups*phases) mixed analysis of variance analyses for thigh muscle co-contraction indices and ratios of UMDH and ULDH.

Variable	Main effect of groups		Main effect of phases		Interaction (groups*phases)	
UMDH	*p*	F (df)	*p*	F (df)	*p*	F (df)
Q:H ratio^ɥ^	0.856	0.16 (2,77)	** < 0.001**	244.35 (1.78,136.90)	0.452	0.91 (3.56,136.90)
Medial Q:H CCI^ɥ^	0.141	2.01 (2,77)	** < 0.001**	118.48 (1.60,122.86)	0.025*	3.14 (3.19,122.86)
Lateral Q:H CCI^ɥ^	0.288	1.27 (2,77)	** < 0.001**	35.30 (1.29,99.50)	0.030*	3.29 (2.59, 99.50)
Medial-to-lateral Q:H CCR^ɥ^	0.342	1.09 (2,77)	** < 0.001**	19.37 (1.70,130.98)	0.094	2.11 (3.40, 130.98)
ULDH						
Q:H ratio^ɥ^	0.696	0.36 (2,77)	** < 0.001**	148.06 (1.57,120.76)	0.124	1.94 (3.14,120.76)
Medial Q:H CCI^ɥ^	0.127	2.12 (2,77)	** < 0.001**	157.05 (1.83,141.21)	0.144	1.77 (3.67,141.21)
Lateral Q:H CCI^ɥ^	0.496	0.71 (2,77)	** < 0.001**	27.32 (1.35,104.16)	0.455	0.86 (2.71,104.16)
Medial-to-lateral Q:H CCR^ɥ^	0.219	1.55 (2,77)	**0.019**	4.36 (1.71,131.79)	0.729	0.469 (3.42,131.79)

Significant values are in [bold].

CCR: cocontraction ratio; Q:H: Quadriceps to hamstring; Q:H CCI: Quadriceps to hamstring cocontraction index; ULDH: unanticipated lateral direction hop; UMDH: unanticipated medial direction hop.

^ɥ^Mauchly’s test of sphericity was significant; therefore, degrees of freedom were adjusted with a Huynh–Feldt correction.

*Significant interactions followed by post hoc tests with a Bonferroni correction did not reveal significant differences between groups for any of the phases; however, there was an increase in the mean (normalized EMG) scores of the groups over time (phase 1–phase 3), more so for patients with ACLR and healthy athletes.

All analyses were performed with log transformed data.

### Medial and lateral Q:H CCIs

For medial and lateral Q:H CCIs (UMDH and ULDH), all groups demonstrated a low CCI in the pre-landing phase with gradually increasing CCIs for the IC and deceleration phases. However, there was a significant interaction between groups and phases for medial and lateral Q:H CCIs for UMDH (*p* ≤ 0.030); however, post hoc tests did not reveal significant differences between groups for any of the phases. Even so, there was an increase in the mean (normalized EMG) scores of the groups through the phases, more so for individuals with ACLR and athletes. The deceleration phase had the highest Q:H CCI value amongst the phases of interest (Table 4). No interaction between groups and phases was evident for ULDH (*p* > 0.050; Table 5).

### Medial-to-lateral Q:H CCR

A significant main effect of phases (but not of groups) was found for UMDH (*p* < 0.001) which implied that the landing phases were different regardless of the group. The IC and deceleration phases had a higher value (muscle co-contraction: medial > lateral) compared to the pre-landing phase for ACLR and control groups (Table 4). Although main effects of phases were significant for ULDH (*p* = 0.019), post hoc comparisons did not reveal significant differences between phases (*p* > 0.050). Nevertheless, a trend similar to UMDH was observed for the phases of land-and-cut maneuver. No significant interaction between groups was found for the medial-to-lateral Q:H CCRs of UMDH and ULDH.

Similar to Q:H ratios, the medial-to-lateral Q:H CCRs demonstrated a low ratio in the pre-landing phase and a high ratio in the IC and/or deceleration phases. In general, a higher medial thigh muscles activity and a relatively lower lateral thigh muscles activity were observed during the deceleration phase of landing. Medial thigh muscles activity was higher than that of the lateral thigh muscles in the IC phase compared to the other phases (Table 4). Regardless of group, the pre-landing phase CCRs were lesser than those of the IC and/or deceleration phases for UMDH and ULDH. However, controls took longer (UMDH: 0.64 ± 0.17 s; ULDH: 0.67 ± 0.17 s) to perform the land-and-cut maneuver compared to those with ACLR (UMDH: 0.56 ± 0.14 s; ULDH: 0.62 ± 0.16 s) and athletes (UMDH: 0.49 ± 0.15 s; ULDH: 0.52 ± 0.15 s).

Box plots of Q:H ratios and medial-to-lateral Q:H CCRs for all three groups have been included as supplementary information.

## Discussion

Our study aimed to describe and compare the EMG activity of the quadriceps and hamstrings muscles during a novel one-leg double-hop test involving a forward hop immediately followed by a diagonal hop performed in medial or lateral direction between ACLR, athlete and control groups. The task, being unanticipated in nature, was designed to mimic sports-specific land-and-cut maneuvers, in which non-contact ACL injuries frequently occur.

The Q:H ratio of all groups exhibited a similar trend for UMDH and ULDH with a particularly high quadriceps activity and a comparatively low hamstring activity in the IC and deceleration phases. On the other hand, hamstrings activity was more dominant than that of the quadriceps in the pre-landing phase (Table 4). A high hamstring activity prior to landing would cause eccentric deceleration of the tibia and prepare the knee for landing with optimal absorption of the forces created by ground contact. The high quadriceps activity with a relatively low hamstring activity immediately after IC could be seen as a risk factor for ACL injury as it increases the anterior translation of the tibia over the femur^18,20,64^. However, a high sagittal plane load alone may not be enough to cause an ACL injury since the risk factors for the injury are multifactorial^20^, also involving other intrinsic and extrinsic factors.

Discordant with our findings, a study by Ford et al. (2011) reported greater quadriceps (rectus femoris, vasti medialis and lateralis) activity compared with the hamstrings (biceps femoris and semitendinosus) in the pre-landing phase for healthy athletes while performing a bilateral drop-vertical jump from a height of 45 cm^65^. Conversely, in agreement with our findings, they did find hamstrings activity to be greater than the quadriceps in the pre-landing phase for drop-vertical jumps performed at lower drop heights (15 and 30 cm). Increased thigh muscle (quadriceps or hamstrings) activity in the pre-landing phase implies preparation for landing^66^ owing to feed-forward control^67^ associated with anticipation of variations in joint movements and forces required for task-specific landing^68^. However, the preferential activation of quadriceps with landing from an increased drop height might be due to increased demand of the task on the knee joint^65^. EMG activity patterns seem to be individual- and task-specific with different recruitment strategies. A recent systematic review did not find significant differences in quadriceps and hamstrings activity onset prior to one-leg landing or decelerating tasks between individuals with ACL injury/ACLR and asymptomatic controls^41^. There might be a lack of difference in EMG onset between thigh muscles during certain tasks which involve deceleration^41^ which warrants further investigation in relation to UMDH/ULDH. However, in the current study, we did not investigate EMG onsets, and the magnitude of activity differed between thigh muscles prior to and during the land-and-cut maneuver of UMDH and ULDH.

For UMDH and ULDH, the medial and lateral Q:H CCIs gradually increased from the pre-landing phase to the deceleration phase (Table 4). This reveals a high contrast for quadriceps and hamstrings activity (vastus medialis vs. medial hamstring and vastus lateralis vs. biceps femoris) in the pre-landing phase that was gradually reduced over the IC and deceleration phases. This imbalance in CCIs occurred because of a greater increased recruitment of the hamstrings compared to the quadriceps for both medial and lateral muscle groups in the pre-landing phase; however, vice versa was true for the IC and deceleration phases. Increased hamstring activity prior to landing reflects preparation for landing^66^ which could be mediated by feed-forward control^67^ in anticipation of joint movements and loads associated with landing^68^. Even so, similar to our findings, quadriceps activity has been reported to be higher than the hamstrings during the deceleration phase of side-step cutting^69^ and jump landing maneuvers^70^. Increased quadriceps activity could increase anterior tibial translation and strain the ACL from 0° to 45° of knee flexion^71^. However, clinicians and also individuals with ACLR should note that sagittal translation of the tibia remains lower in closed kinetic chain exercises compared to open kinetic chain exercises^72^ incorporated in rehabilitation.

The medial-to-lateral Q:H CCRs were not significantly different between groups. The IC and deceleration phases had a higher value than the pre-landing phase for ACLR and control groups. The medial-to-lateral Q:H CCRs (Table 5) indicate predominant coactivation of the medial thigh muscles in relation to that of the lateral group. This might cause knee adduction and thus counteract external knee abduction moments^64^ during the IC and deceleration phases. These findings seem to be concordant with the observed knee adduction angles during the deceleration phase (UMDH: ACLR, 7.36° ± 4.93°; athletes, 7.57° ± 6.39°; controls, 7.62° ± 4.43°; ULDH: ACLR, 6.85° ± 4.70°; athletes, 10.74° ± 7.32°; controls, 9.51° ± 6.64°; methods for kinematic analysis are reported elsewhere^31,32^). However, we found weak insignificant Pearson correlations (*r* < 0.30; *p* > 0.05) between medial-to-lateral Q:H CCR and knee adduction angles for the deceleration phase of the land-and-cut maneuver for all three groups for both (medial and lateral) direction of hops. Although these correlations addressed the frontal plane mechanics, it is important to consider the relationship between multiplanar movement combinations and CCIs/CCRs of the knee in subsequent studies. Whether medial-to-lateral Q:H CCR, medial Q:H CCI or medial quadriceps and hamstring EMG amplitudes would be associated with a significant variation in the frontal plane knee angles, and external or internal moments during different phases of landing warrant further detailed investigation in the future studies. We recommend incorporating various covariates (*e.g.,* most importantly ACL injury status, comorbidities [e.g. osteoarthritis, concomitant ligament injuries, etc.], kinesiophobia, dynamic knee stability measures, physical activity level, self-reported outcome measures, knee laxity, time since surgery/injury) while analyzing the relationship between multiplanar knee angles/moments and thigh muscle CCIs/CCRs or temporal parameters (EMG onsets and duration of co-contraction of muscle pairs).

Prior training might help with neuromotor planning and anticipatory contraction of the lower limb muscles that control and stabilize the knee^42^. Nevertheless, there is a lack of consensus regarding an optimal neuromuscular training to be implemented following ACLR^73^. Zebis et al. found that neuromuscular training aimed at preventing non-contact ACL injuries might help to increase selective activation of the medial hamstrings to decrease external rotation and abduction of the knee during instep and side-cutting maneuvers^47^. In the current study, we documented only the current physical activity levels of the healthy controls based on the IPAQ and Tegner scores; therefore, their prior experience with cutting maneuvers or knee demanding activities was not fully explored. It cannot be ruled out whether all three groups (ACLR, athletes and controls) may have experienced some form of training that resulted in a lack of difference between groups.

Some methodological considerations pinpointing limitations of the study are summarized below. Performing UMDH and ULDH in a controlled laboratory environment will not exactly emulate sports-specific cutting maneuvers endangering ACL integrity. Though our participants were asked to perform UMDH/ULDH as quickly as possible by reacting to the visual cue, this may still have taken a longer time than that to perform a cutting maneuver in a real sporting situation.

We assumed that a difference in males:females ratio (males ≤ 26% in each group) might not affect between-group comparisons owing to that the proportion was similar in all groups. Despite a statistically significant difference in age of participants (ACLR vs. athletes), they were not expected to have any age-related changes of physical attributes affecting thigh muscle activity. Participants (aged 17–34 years) in all groups were screened for musculoskeletal impairments by an experienced physiotherapist prior to data collection.

As we have included participants with a hamstring graft in the ACLR group, whether it would adversely affect medial hamstring activity remains ambiguous. Following a semitendinosus graft the regeneration of the semitendinosus tendon after 1–3 years^74,75^ could result in a more proximal insertion of the distal tendon (⁓4 cm) and a decreased muscle moment arm. This might lead to an increase in EMG activity of the medial hamstring because of an increased motor unit activation to produce more force to match an equal amount of muscle torque^76^. Individuals with ACLR had the surgery 7 to 129 months (median 18 months) prior to their participation in the study and the extent of regeneration of the semitendinosus tendon and associated changes in EMG co-contraction patterns might be variable among the participants (undergoing rehabilitation vs. returned to functional activities/sports). A longitudinal prospective cohort study thoroughly addressing the influence of time aspect on such outcomes is warranted.

A novel standardized rebound side-hop recently demonstrated that individuals with ACLR with a high fear of re-injury display significantly higher thigh muscle co-contraction when compared to ACLR individuals with a low fear of re-injury and asymptomatic controls^77^. Our knee-challenging one-leg double-hop test might be even more suitable to investigate objective measures of movement coordination in relation to fear of re-injury, which is one of the major reasons preventing return to sports^78^.

Future studies can also compare variations in our double-hop test and subsequent changes in EMG co-contraction patterns of individuals with and without ACL injuries; for instance, incorporating penultimate contact with the non-dominant foot followed by final foot contact with the dominant foot or vice versa at varying angles of direction change (45°, 90°, etc.)^79^. We recommend recording gastrocnemius (an antagonist of the ACL)^80^ activity along with the thigh muscles and analyze the relationship of EMG to knee angles and moments in the future studies. Comparing unanticipated hops with pre-planned hops is further warranted.

## Conclusion

An increased quadriceps activity compared to that of the hamstrings, accounting for a higher Q:H ratio, was found during the IC and deceleration phases compared to the pre-landing phase of the land-and-cut maneuver (UMDH/ULDH) for all groups. All groups, irrespective of ACL injury or physical activity level, showed a relatively low imbalance in the medial and lateral Q:H CCIs for the pre-landing phase compared to the IC and deceleration phases of landing. However, controls took a longer time to complete the task compared to individuals with ACLR and elite athletes. Overall, the findings indicate predominant co-contraction of the medial thigh muscles over the lateral group; if such co-contraction patterns cause knee adduction during the deceleration phases of the land-and-cut maneuver needs further substantiation. Our study results would be useful for clinicians and researchers investigating the role of thigh muscle activation patterns in augmenting or mitigating ACL injury risk during unanticipated one-leg landing tasks.

## Supplementary Information

Supplementary Information.

## Data Availability

Data are available at reasonable request.
