# Supplementary material for: Thigh muscle co-contraction patterns in individuals with anterior cruciate ligament reconstruction, athletes and controls during a novel double-hop test
Source: Sci Rep. 2022 May 19;12:8431. doi: 10.1038/s41598-022-12436-6 (PMC9119948; doi:10.1038/s41598-022-12436-6)

## **Supplementary Information:**

## Box plots showing median and interquartile range values of Quadriceps-to-Hamstring (Q:H) ratio and medial-to-lateral Q:H co-contraction ratios calculated for three phases of landing (100 ms prior to landing [pre-landing], initial contact and deceleration phases) during unanticipated medial diagonal hop (UMDH) and unanticipated lateral diagonal hop (ULDH) in individuals with ACL reconstruction, elite athletes and controls.
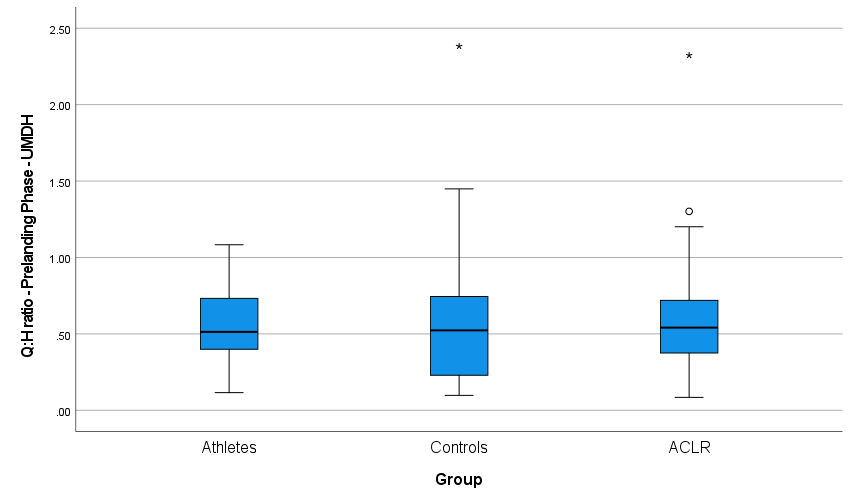


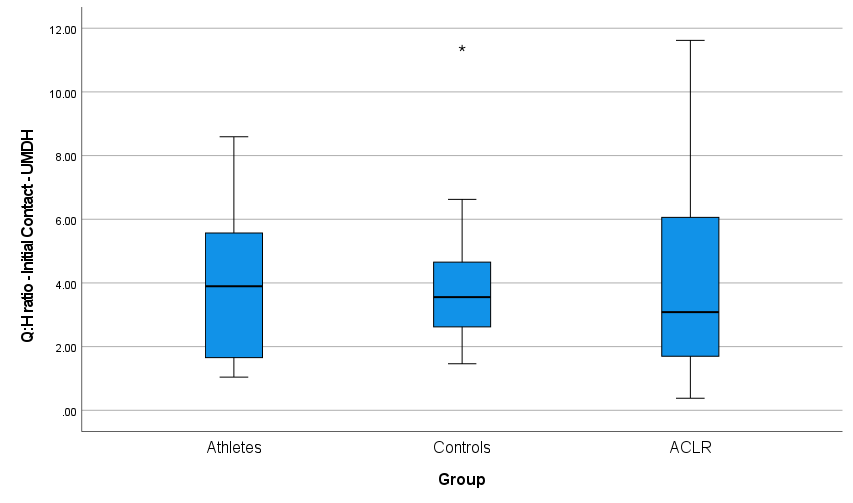


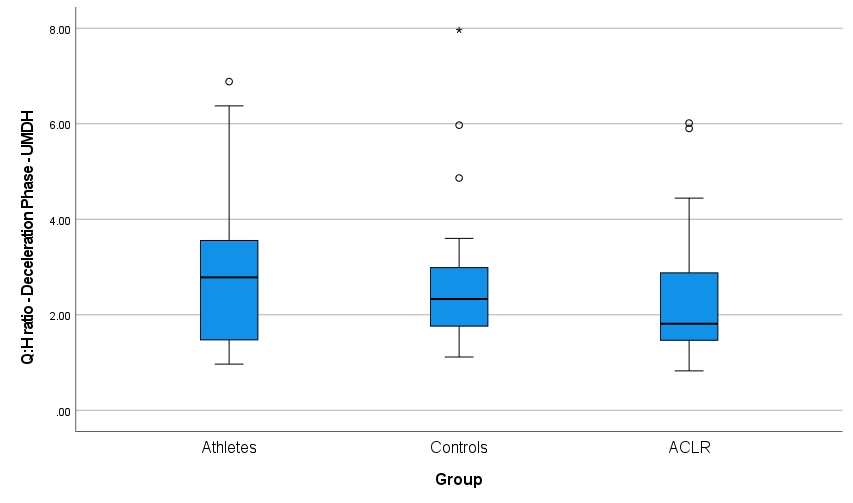


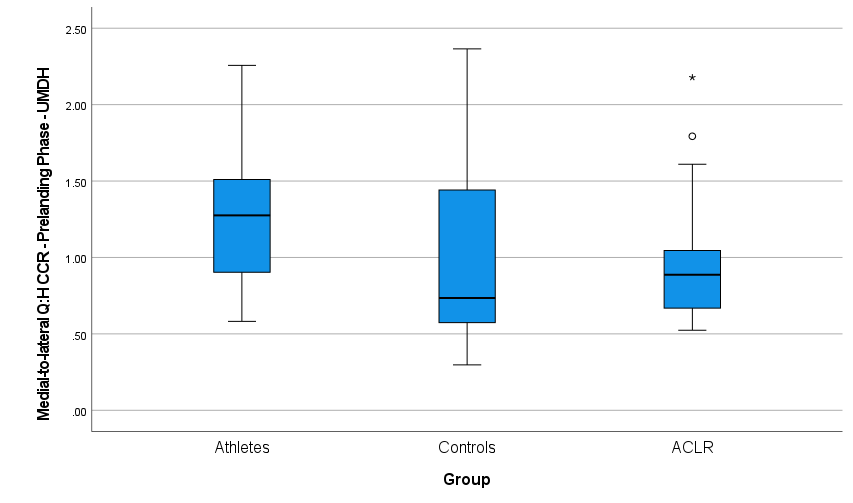


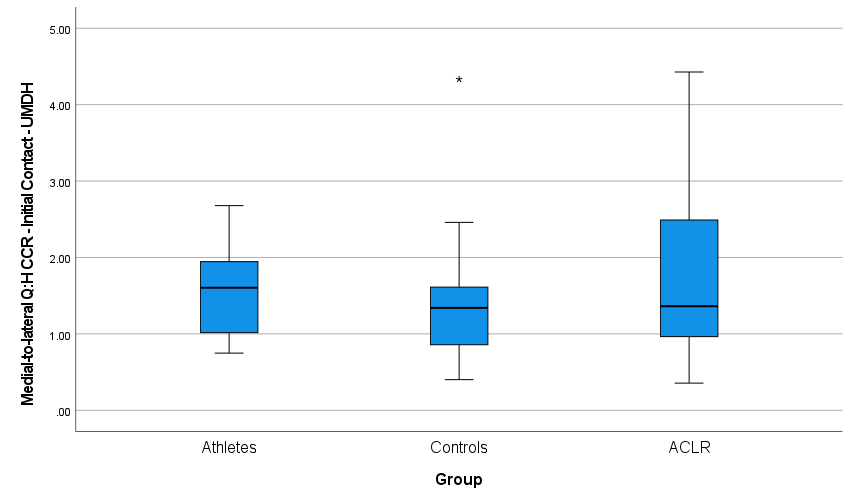


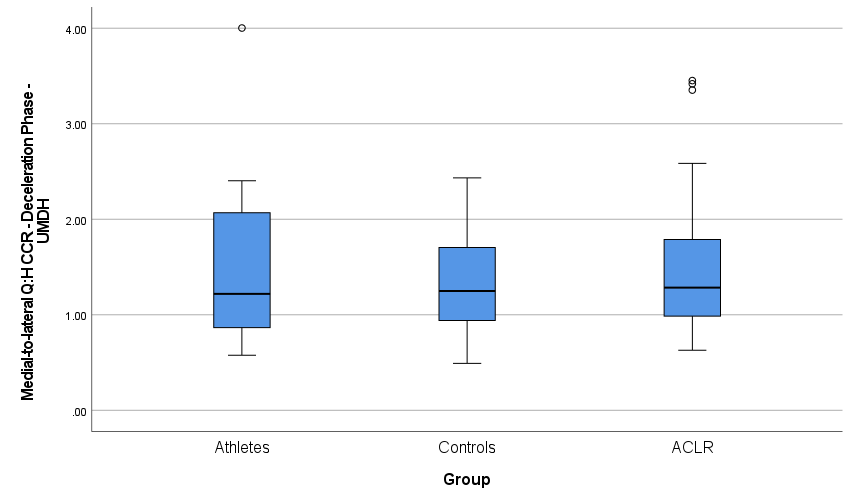


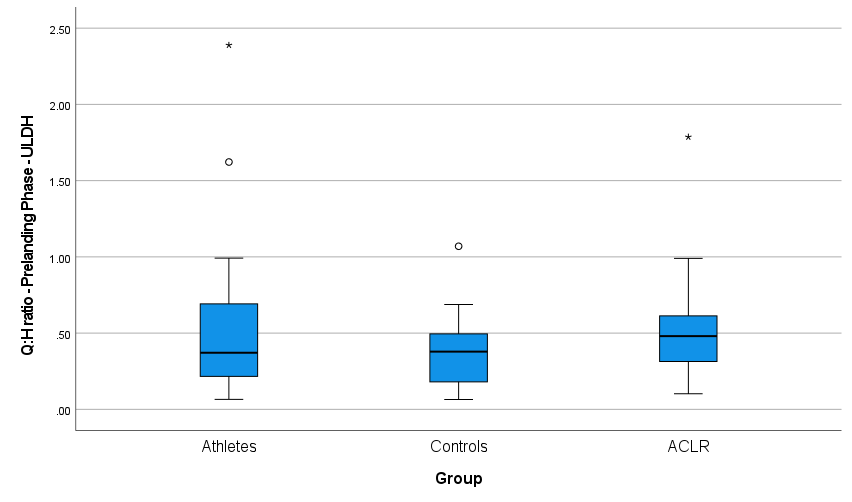


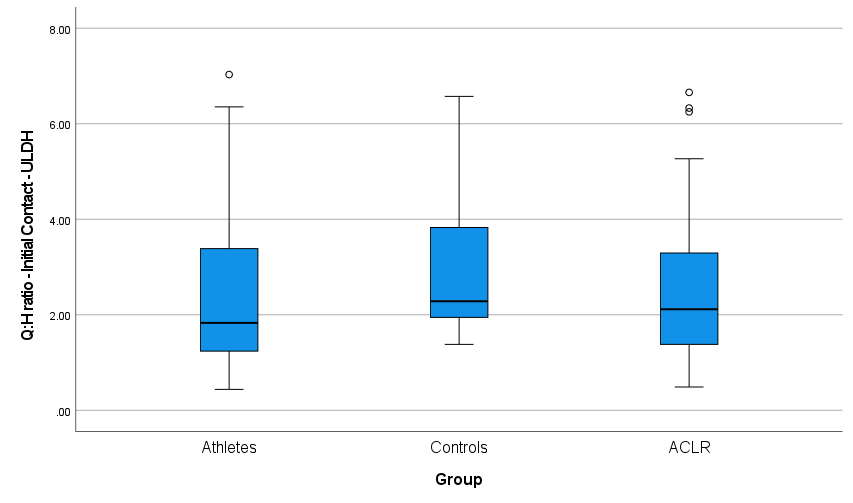


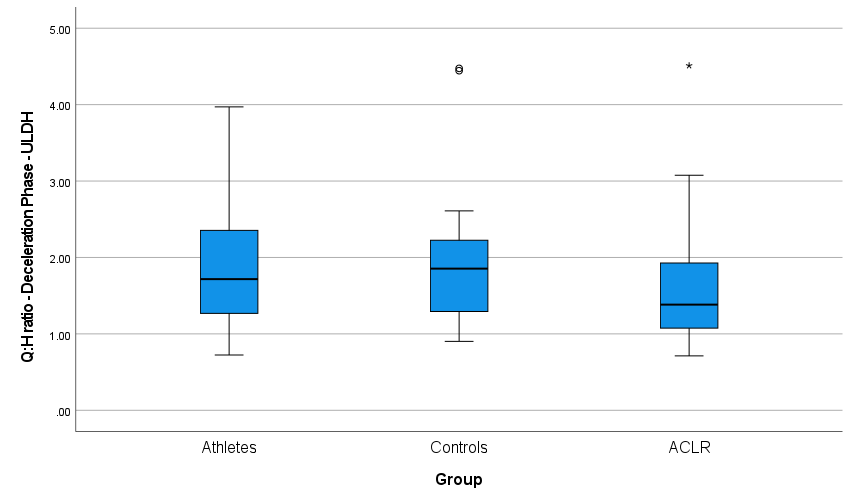


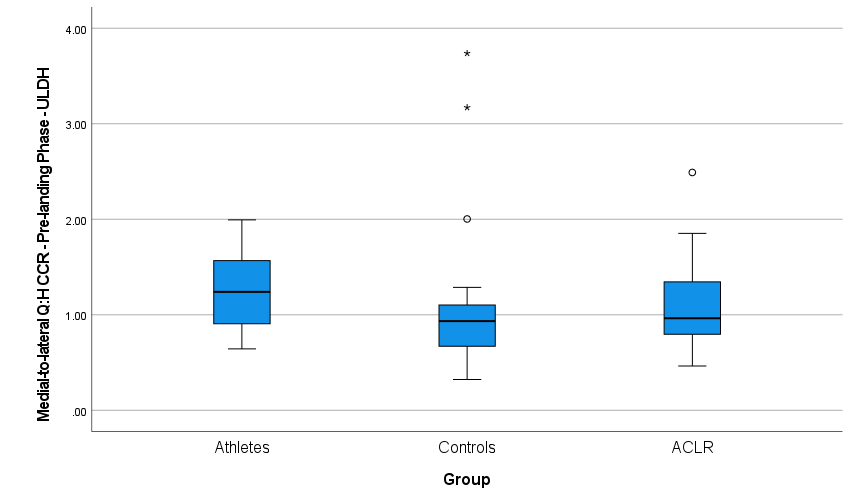


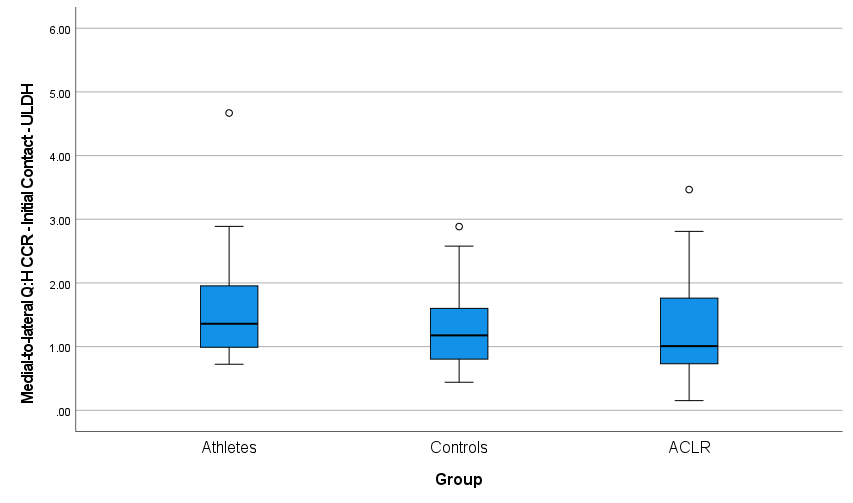


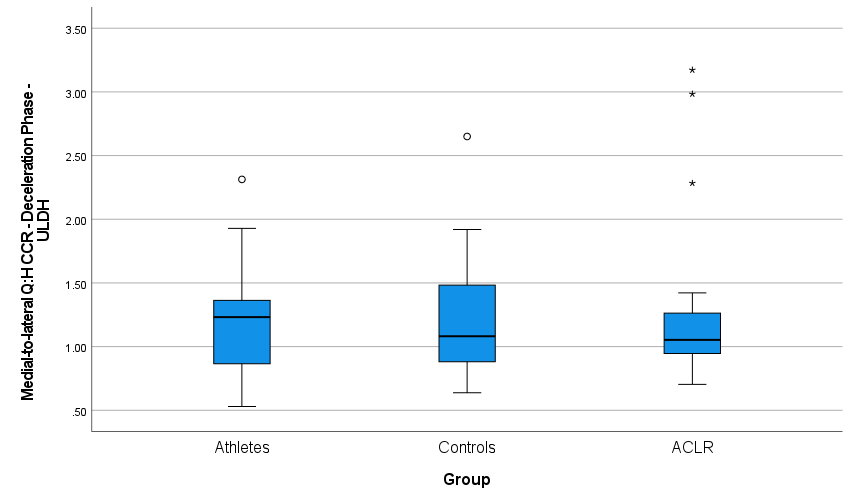

Supplement: Supplementary file 1 — Supplementary Information. [file 41598_2022_12436_MOESM1_ESM.docx]
